# Sclerosing stromal tumor of the ovary in a postmenopausal woman with estrogen excess: A case report

**DOI:** 10.1097/MD.0000000000018171

**Published:** 2019-11-22

**Authors:** Zhiyi Zhao, Li Yan, Hongtao Lv, Hongtao Liu, Fengnian Rong

**Affiliations:** a Department of Gynecology and Obstetrics; b Department of Pathology, Shandong Provincial Qianfoshan Hospital, The First Affiliated Hospital of Shandong First Medical University, Jinan, Shandong, China.

**Keywords:** estrogen excess, postmenopausal bleeding, sclerosing stromal tumor

## Abstract

**Rationale:**

Sclerosing stromal tumor (SST) of the ovary is rare. We describe the first case of ovarian SST with estrogen excess with both clinical and serological evidence in a postmenopausal woman.

**Patient concerns:**

A 70-year-old female who referred menopause 14 years ago was admitted with postmenopausal bleeding for 3 months. Ultrasonography revealed thickened endometrium of 6 mm and no adnexal masses. An elevated serum estradiol level of 49.78 ng/L was revealed in laboratory examination with normal ranges less than 27.25 ng/L in postmenopausal women.

**Diagnoses:**

The final histology diagnosis is SST of left ovary and endometrial hyperplasia with polyps.

**Interventions:**

Laparoscopic hysterectomy and bilateral salpingo-oophorectomy were performed and a solid tumor with a diameter of 3 cm × 2 cm from the left ovary was found intraoperatively.

**Outcomes:**

Three days after removal of the tumor, the serum estrogen level was decreased to normal which indicated the estrogen activity of the tumor.

**Lessons:**

To the best of our knowledge, it is the first reported case of ovarian SST with estrogen excess with both clinical and serological evidence. The present case illustrates the necessity to consider the rare possibility of ovarian SST as a cause for estrogen excess leading to postmenopausal bleeding. Hormonal evaluation (estrogens, androgens) should be considered in women with postmenopausal bleeding regardless of imaging examination.

## 1 Introduction

Sclerosing stromal tumor (SST) of the ovary is rare and predominantly observed in young women of the second and third decades. Most patients with SST present with menstrual irregularities, pelvic pain, and nonspecific symptoms associated with presence of a pelvic mass (mean diameter of about 10 cm).^[1]^ Ovarian SST is hormonally inactive in the majority of previously reported cases. A few reports demonstrated that it was associated with higher serum androgens and cause virilization. Rarely, it was shown to be related to estrogen activity; however, no case of ovarian SST has been reported for elevated serum estrogen level with serological confirmation. In the present manuscript, details of a rare case study including an ovarian SST in a postmenopausal female with estrogen excess are described. To the best of our knowledge, it is the first reported case of ovarian SST with estrogen excess with both clinical and serological evidence.

## 2 Case presentation

In July 2017, a 70-year-old Chinese woman presented at the Shandong Provincial Qianfoshan Hospital, China with postmenopausal bleeding (PMB) for 3 months. She referred menopause 14 years ago. Following admission, ultrasonography revealed thickened endometrium of 6 mm and no adnexal masses. Cervical smear test result was normal. An elevated serum estradiol level of 49.78 ng/L was revealed in laboratory examination with normal ranges less than 27.25 ng/L in postmenopausal women. Serum testosterone, progesterone, calcium 125, liver function, and thyrotropin were all normal. No virilization was observed. She denied any chronic disease or any use of hormonal replacement therapy.

**Figure 1:**
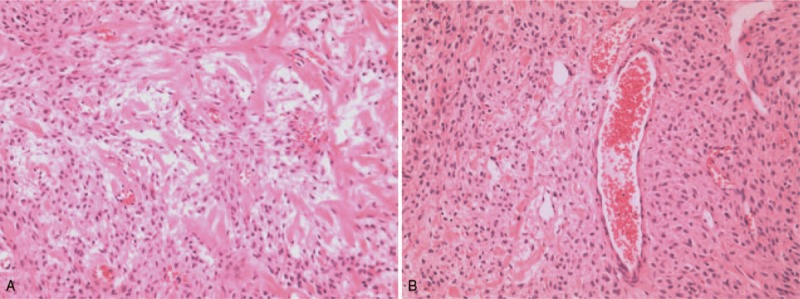
The ovarian mass was stained with hematoxylin and eosin (magnification, ×100). (A) Characteristic cellular pseudolobular pattern separated by edematous fibrous bands and increased vascularity. (B) Dilated thin-walled blood vessels.

Owing to the postmenopausal status of the patient and her unwillingness of keeping uterus and bilateral accessories, laparoscopic hysterectomy and bilateral salpingo-oophorectomy were performed as therapy for a hidden ovarian tumor and presumed endometrial malignancy. Intraoperatively, a solid tumor with a diameter of 3 cm × 2 cm from left ovary was found. The frozen section was reported as benign, suggestive of a sex cord stromal tumor of the left ovary and endometrial hyperplasia with polyps. The immunohistochemistry result included positive staining for alpha-inhibin and negative staining for CD10 of the left ovary tumor. The final histology diagnosis is SST of left ovary and endometrial hyperplasia with polyps (Fig. 1).

Three days after the surgery, the serum estradiol level decreased to less than 5 ng/L, demonstrating the normalization of estrogen levels. Her postoperative course was smooth and she was discharged on the fifth day after surgery. There was no evidence of recurrence during follow-up until now. The study was approved by the Ethics Committee of Shandong Qianfoshan Hospital and the patient has provided informed consent for publication of the case.

## 3 Discussion

Ovarian SST, which was first described by Chalvardjian and Scully in 1973, is a rare ovarian neoplasm accounting for less than 5% of sex cord-stromal tumors. SST is attributable to theca cell-fibrous tumor subtypes of ovarian sex cord-stromal tumor according to the World Health Organization-2003 classification. Macroscopically, they are usually grey solid tumors with smooth external surface intermingled with a focal sallow discoloration.^[2]^ Microscopically, SST is characterized by the presence of pseudo-lobulated cellular areas, which were separated by densely collagenous, edematous or myxoid tissue and increased vascularity.^[3]^ Immunohistochemistry is useful for differential diagnosis.

Ultrasonography is the first choice for diagnosis of ovarian SST; however, it cannot predict the presence of ovarian SST preoperatively. Ovarian SST could be too small to been detectable. Besides, evaluations could be limited by gas in the duodenum which blocks sound waves and abdominal subcutaneous adipose tissue which attenuates the sound beam. Computed tomography or magnetic resonance imaging is helpful but it is still difficult to diagnose ovarian SST before surgery by imaging examinations.^[4]^ The definitive diagnosis is based on accurate histologic analysis.^[3]^

Histologically, SST is characterized by the presence of pseudolobulated cellular areas, which were separated by densely collagenous, edematous or myxoid tissue and increased vascularity composed of thin-walled blood vessels.^[4]^ Several immunohistochemical markers were investigated in ovarian SST. Inhibin has been shown to be consistent positive and be a useful marker for ovarian sex cord stromal tumors. Besides, ovarian SST cells were reported to be also consistent positively immunostained for vimentin and vascular endothelial growth factor; and have negative reactivity for S-100 protein and epithelial markers.

Ovarian SST is hormonally inactive in the majority of previously reported cases. A few reports demonstrated that it was associated with higher serum androgens and cause virilization.^[1]^ Rarely, it was shown to be related to estrogen activity. Damjanov et al reported a case with urinary excretion of estrogens and androgens reduction after removal of the tumor.^[5]^ Two cases were reported to have clinical features suggestive of estrogen activity from the tumor.^[6]^ No case of ovarian SST has been reported for elevated serum estrogen level with serological confirmation. Our case presented with PMB, endometrial hyperplasia and elevated serum estradiol level, indicating estrogen excess. After removal of the tumor, the serum estrogen level was decreased to normal in 3 days which indicated the estrogen activity of the tumor. To the best of our knowledge, it is the first reported case of ovarian SST with estrogen excess with both clinical and serological evidence.

Elevated or prolonged serum estrogen stimulation on endometrium may lead to endometrial hyperplasia or even carcinoma, which is a hormone-dependent tumor associated with high circulating estrogen level. Endometrial adenocarcinoma concomitants with ovarian SST has also been described, although no apparent evidence of active steroid hormone secretion was detected by plasma analysis.^[7]^ Therefore, early diagnosis and treatment of estrogen excess is important.

PMB is defined as uterine bleeding occurring after at least 12 months of amenorrhea. It is a common clinical condition with an incidence of 10%. Women with PMB have a 10% to 15% risk of having endometrial carcinoma and approximately 90% of patients with endometrial carcinoma presents with PMB as the only complaint.^[8]^ Therefore, prompt and accurate evaluations are required to exclude cancer or precancerous lesions of the endometrium for women with PMB. The main reason of PMB is due to an intrauterine source, rarely, it may be related to ovarian tumors. Transvaginal ultrasound scanning and endometrial biopsy, the first line of investigation of PMB,^[9]^ cannot accurately locate the lesion in ovary. The preset case illustrates the necessity to consider the rare possibility of ovarian SST as a cause for estrogen excess leading to PMB. Hormonal evaluation (estrogens, androgens) should be considered in women with PMB for excluding ovarian tumors with hormone activity regardless of imaging examination.

Ovarian SST is considered to be benign except for 1 case with low grade malignancy reported.^[10]^ Surgical removal of the tumor is curative, and no local or distant recurrences have been reported until now.

In conclusion, we presented the first case of ovarian SST with estrogen excess with both clinical and serological evidence in a postmenopausal woman. The present case illustrates the necessity to consider the rare possibility of ovarian SST as a cause for estrogen excess leading to PMB. And hormonal evaluation (estrogens, androgens) should be considered in women with PMB for excluding ovarian tumors with hormone activity regardless of imaging examination.

## Author contributions

**Investigation:** Hongtao Lv, Hongtao Liu.

**Writing – original draft:** Zhiyi Zhao, Li Yan.

**Writing – review and editing:** Zhiyi Zhao, Fengnian Rong.
